# Development of a cost-effective medium for enhanced biomass-associated fucoxanthin and bio-silica yields of diatom (*Melosira nummuloides*)

**DOI:** 10.71150/jm.2512005

**Published:** 2026-02-28

**Authors:** Ve Van Le, Nam-Ho Lee, Gyung-Min Go, Somi kim Cho, Man-Young Jung, Sang-Ah Lee

**Affiliations:** 1 Faculty of Biotechnology, College of Applied Life Sciences, Jeju National University, Jeju 63243, Republic of Korea; 2 JDK Bio, Jeju 63296, Republic of Korea; 3 Interdisciplinary Graduate Program in Advance Convergence Technology and Science, Jeju National University, Jeju 63243, Republic of Korea; 4 Bio-Health Materials Core-Facility Center, Jeju National University, Jeju 63243, Republic of Korea; 5 Department of Science Education, Jeju National University, Jeju 63243, Republic of Korea

**Keywords:** MOM-FS, fucoxanthin, *Melosira nummuloides*, biogenic silica, diatom

## Abstract

Fucoxanthin has gained attention for its beneficial effects, including anti-cancer, anti-obesity, and anti-inflammatory activities. A benthic marine diatom *Melosira nummuloides* is a promising candidate for fucoxanthin production. Nevertheless, industrial-scale cultivation remains constrained by suboptimal growth performance and the lack of species-tailored media. This study aimed to develop a cost-effective medium for enhancing biomass and fucoxanthin production in *M. nummuloides* by modifying the conventional F/2 medium based on species-specific intracellular nutrient stoichiometry. The cellular molar N:P:Si ratio of *M. nummuloides* was identified as 13:1:12.3. Despite nitrogen reduction by 36.13% relative to F/2 medium, *M. nummuloides* cultivated in the *Melosira*-Optimized Medium using Fumed Silica (MOM-FS) was well grown, achieving biomass concentration of 261 mg/L on day 4—approximately 1.21-fold higher than that obtained with F/2. In addition, MOM-FS enhanced biomass-associated fucoxanthin yield by 10.3% and biogenic silica yield by 20.8% relative to the F/2. The use of MOM-FS reduced total medium costs by 28.3%, fucoxanthin production cost by 36.8%, and bio-silica production cost by 28.3%. Overall, these findings indicate that the cost-effective medium developed here provides a practical, efficient, and economically viable framework for large-scale cultivation of *M. nummuloides* and the co-production of fucoxanthin and bio-silica.

## Introduction

Fucoxanthin is one of the most abundant carotenoids and has considerable potential for nutraceutical and pharmaceutical applications, particularly in promoting human health and managing diseases (Kumarasinghe and Gunathilaka, 2024). Because fucoxanthin remains a key compound with potent anti-cancer, anti-obesity, and anti-tumor properties, its global demand continues to rise (Lourenço-Lopes et al., 2021). However, due to its complex molecular structure, fucoxanthin cannot be synthesized via petrochemical routes and is therefore primarily extracted from marine macroalgae (Pajot et al., 2022). The low fucoxanthin content (0.02–0.58 mg/g of fresh cell weight) (Kanazawa et al., 2008; Kim et al., 2011) and slow growth of macroalgae limit their suitability for industrial-scale fucoxanthin production (Sun et al., 2023).

Compared to macroalgae, microalgae have a higher growth rate and fucoxanthin content, gaining attention as promising alternative fucoxanthin producers (Yoshida et al., 2023). *Melosira nummuloides*, belonging to the family *Melosira*ceae, is a benthic marine diatom commonly found coastal zone associated with Jeju lava seawater, Korea. This species has been also recognized for its diverse pharmacological activities, such as anticancer, antioxidant, and anti-inflammatory (Cuong et al., 2023; Do et al., 2025). *M. nummuloides* has been identified as a rich source of polyunsaturated fatty acids and fucoxanthin (Kim et al., 2024). However, the commercial utilization of *M. nummuloides* remains limited, primarily due to its relatively low biomass yield and the absence of an optimal growth medium (Kim et al., 2023).

The primary challenge in the commercial production of fucoxanthin lies in its inherently high production cost, which is attributed to the difficulty of chemical synthesis and the limited scale-up potential of microalgal mass cultivation (Budiarso et al., 2025). The growth medium of *M. nummuloides* is a significant contributor to the final cost of bioprocess (An et al., 2024b). The F/2 medium—one of the most widely used culture media for marine microalgae—originally from the F medium developed for diatoms such as *Cyclotella nana* and *Detonula confervacea* (Guillard and Ryther, 1962). The original F medium was formulated as enriched seawater containing macronutrients (nitrate, phosphate, and silicate), trace metals, and vitamins to support phytoplankton growth. Later, all nutrient concentrations were reduced by half, establishing the F/2 medium, to minimize nutrient toxicity and bacterial contamination while maintaining sufficient algal growth (Guillard, 1975). Although the F/2 medium is well-suited for laboratory cultivation of diverse marine microalgae, its cost makes it economically impractical for large-scale production (Faé Neto et al., 2018). To reduce bioprocess costs, several studies have explored further dilution of the F/2 medium (e.g., F/3 or F/4 formulations); however, such media can induce nutrient limitation and decrease biomass productivity (Kim et al., 2023).

Accordingly, developing a cost-effective medium is essential for large-scale *M. nummuloides* to enhance fucoxanthin biosynthesis and biomass accumulation without compromising yield. Based on the nutrient requirements for optimal microalgal growth, culture-medium components can be classified into macronutrients (C, H, O, N, and P), required at relatively high concentrations, and micronutrients (e.g., cobalt, zinc, manganese, and barium), needed only at trace levels (≈ mg/L or lower) (Daneshvar et al., 2021). Notably, in F/2 medium, the nitrogen source accounts for 48.59% of the total cost and is therefore considered the primary target for cost reduction (An et al., 2024b).

The elemental stoichiometry of marine plankton, expressed as C:N:P ≈ 106:16:1 and known as the Redfield ratio, is often regarded as the optimal nutrient balance for diatom growth, while N:Si:P ≈ 15:6:1 is also considered important (Redfield, 1960). However, this stoichiometry varies among taxa and should be determined for each species to maximize biomass production (Carnicer et al., 2022; Grubišić et al., 2024; Redfield, 1958). The cellular N:Si:P molar ratio of *M. nummuloides* has not yet been identified. Prior work has focused on general culture conditions for the growth of *M. nummuloides*—temperature, salinity, light intensity, and nutrient proportions—with reported optimal N:P ratios ranging from 15:1 to 50:1 (Ji et al., 2013; Kim et al., 2023). However, no earlier studies have tailored F/2 medium based on species-specific intracellular N:P:Si ratios and evaluated the cost effectiveness of culture media for fucoxanthin production.

To enable commercial scale fucoxanthin production using *M. nummuloides*, this study developed a cost-effective culture medium by reformulating the conventional F/2 medium based on species-specific intracellular N:P:Si stoichiometry. The modified medium is expected to enhance algal biomass productivity and thereby support the large-scale fucoxanthin production.

## Materials and Methods

### Microalgal strain and culture conditions

*M. nummuloides* strain JDK001 was obtained from the Marine Bio-Resource Information System (MBRIS) of the National Marine Biodiversity Institute of Korea (MABIK). The Jeju lava seawater —a type of saline groundwater formed by the natural infiltration of seawater through volcanic bedrock— was filtered through a 0.22 μm bottle-top vacuum filter (CLS430015, Corning Inc., USA). The F/2 medium was prepared by dissolving F/2 broth (MB-F0701, MBcell, Korea) in Jeju lava seawater (An et al., 2024b). The mixture was heated until completely dissolved and then sterilized by autoclaving at 121°C for 15 min. After the F/2 medium was cooled to 45–50°C, F/2 trace metal solution (MB-F0766, MBcell, Korea) and vitamin solution (MB-F0767, MBcell, Korea) were added to the medium. *M. nummuloides* was cultivated in 1 L polycarbonate Erlenmeyer flasks (431255; Corning Inc., USA) containing 400 ml of medium. Cultures were incubated at 24°C with agitation at 120 rpm under continuous white LED illumination at an intensity of 60 µmol/m^2^/s.

### Normalization of intracellular nutrient ratios

The cell number of *M. nummuloides* was counted using a disposable hemocytometer (C-Chip, DHC-N01-5, iNCYTO, Korea) under a phase-contrast microscope (Axiovert 5 digital, ZEISS, Germany, in Bio-Health Materials CoreFacility, Jeju National University). For intracellular nutrient analysis, cells were harvested after 4 days of cultivation by centrifugation at 5,000 × g for 10 min and lysed using 1 M sodium hydroxide solution (7571-4400, Daejung, Korea). Total nitrogen (TN) and total phosphorus (TP) concentrations were measured using water quality analysis kits (10213-TN and 10612-TP; C-mac, Korea) according to manufacturer’s instructions. Silica (Si) concentration was determined using a high-range silica photometer (HI 97770; Hanna Instruments, USA). Measured nutrient concentrations were normalized to cell counts to calculate the intracellular N:P:Si ratio.

### Development of modified culture media

The modified culture media were derived from the standard formulation of F/2 medium by adjusting nitrogen concentration to match the intracellular N:P:Si stoichiometry of *M. nummuloides* (Table 1). In addition, because the silicate source in F/2 medium—sodium metasilicate nonahydrate (Na_2_SiO_3_·9H_2_O; Daejung, Korea)—is not cost-effective for large-scale applications, two media were developed: (i) *Melosira*-Optimized Medium using Meta-Sodium Silicate (MOM-MS), which employs Na_2_SiO_3_·9H_2_O as in F/2; and (ii) *Melosira*-Optimized Medium using Fumed Silica (MOM-FS), which uses fumed silica powder (SiO_2_; Sigma-Aldrich, USA) as a lower-cost alternative (Table 1). For MOM-FS medium, the fumed silica was converted to soluble sodium silicate by reaction with sodium hydroxide (NaOH; Daejung, Korea; cat. 7571-4400) prior to incorporation into the medium. The composition of the culture media is presented in Table 1. In the modified media, the concentrations of phosphorus, trace metals, and vitamins were identical to those in the F/2 medium, while the nitrogen concentration was reduced by 36.13% compared with the standard F/2 formulation.

### Determination of cell density and biomass

*M. nummuloides* cells were inoculated at an initial concentration of 0.1 g/L and incubated at 120 rpm (JSSI-4CM, JSR, Korea) for 4 days at 24°C. Dry cell weight (DCW) was determined by filtering 10 ml of culture through glass fiber filters (Whatman GF/C^TM^, 1822-025, Cytiva, UK), followed by drying at 105°C for 4 h. The filtrate was used to measure N, P, and Si concentrations. Chain length was assessed by microscopic observation. The growth rate (µ) and doubling time (τ) of *M. nummuloides* were calculated according to the following equations:


(1) $$\mathrm{Growth}\;\mathrm{rate}\;(µ)\;=(\ln\;N_{t}-\ln\;N_{0})/(t-t_{0})$$

(2) Doubling time (τ) =ln 2/μ

where N_0_ and N_t_ are the cell concentration at t_0_ and the end (t) of the selected time, respectively.

The removal rates (R) of nitrogen, phosphorus, and silica and the specific rate of substrate removal (R_xi_) were calculated using the following equations (Yang et al., 2016):


$$\mathrm{Removal}\;\mathrm{rates}\;(R)\;=(S_{0}\;-\;S_{i})\;/\;(t_{i}\;-\;t_{0})\;\mathrm{Specific}\;\mathrm{rate}\;\mathrm{of}\;\mathrm{substrate}\;\mathrm{removal}\;(R_{\mathrm{xi}})\;=\;R/\mathrm{dry}\;\mathrm{cell}\;\mathrm{weight}$$

where S_0_ (mg/L) represents the initial concentration of nutrients at time t_0_ (day), and S_i_ (mg/L) is the nutrient concentration at time t_i_ (day).

### Extraction and quantification of fucoxanthin and biosilica

Fucoxanthin was extracted from *M. nummuloides* cells using methanol (100%, 5558-2304, Daejung, Korea) followed by ultrasonic extraction (Bárcenas-Pérez et al., 2021). Fucoxanthin concentration was measured using high-performance liquid chromatography (HPLC) equipped with a C18 reverse-phase column, employing a methanol-water mobile phase at a flow rate of 1 ml/min and detection at 450 nm (Foo et al., 2016). Detection was carried out at 450 nm, and quantification was performed based on a calibration curve prepared with fucoxanthin standard (Supelco, 16337, Sigma-Aldrich, USA) dissolved in methanol (Jaswir et al., 2013). Bio-silica was extracted by suspending cells in 1 M sodium hydroxide solution at room temperature (Rao et al., 2022). The silica content was quantified using a silica ion colorimeter (HI 97770; Hanna Instruments, USA). The production yields of fucoxanthin and bio-silica were calculated by multiplying their respective contents (per unit DCW) by the total DCW of the culture.

### Statistical analysis

All experiments were performed in biological triplicate. One-way analysis of variance (ANOVA) was performed using R version 4.5.1 (R Foundation for Statistical Computing, Vienna, Austria), with statistical significance defined as *p* < 0.05. When the ANOVA indicated significant effects, Tukey’s Honestly Significant Difference (HSD) post-hoc test was used to determine pairwise differences among groups. Significant differences were denoted by different letters, and error bars represent the standard error of the mean.

## Results and Discussion

### Intracellular nutrient content and N:P:Si ratio normalization of *M. nummuloides*

**Fig. 1.**
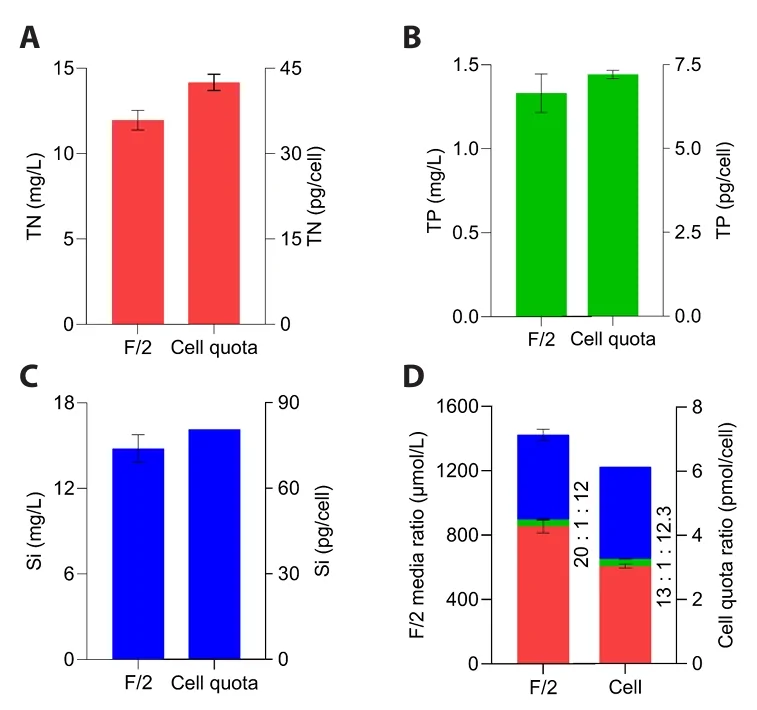
Cellular and medium nutrient composition. (A) Total nitrogen content of *M. nummuloides* cells and the F/2 medium. (B) Total phosphorus content of *M. nummuloides* cells and the F/2 medium. (C) Silica content of *M. nummuloides* cells and the F/2 medium, and (D) N:P:Si ratios of *M. nummuloides* cells (cell quota ratios). Data represent mean values of three biological replicates, with error bars indicating standard error (SE).

Generally, diatom growth was mainly controlled by three macronutrients — N, P, and Si (Bandyopadhyay and Biswas, 2021) — which are therefore supplied at the highest concentrations in the F/2 medium (Grubišić et al., 2024). N and P are crucial for synthesizing structural cellular components such as nucleic acids, proteins, vitamins, and pigments, while Si is primarily used for building their siliceous cell walls (Gilpin et al., 2004). Although the elemental stoichiometry of marine phytoplankton is typically represented by the Redfield ratio (C:N:P ≈ 106:16:1), diatoms generally exhibit an extended ratio including silicon (N:Si:P ≈ 15:16:1) to reflect their requirement for silica frustule formation. However, this stoichiometry varies substantially among taxa and must be determined specifically to achieve optimal biomass production (Carnicer et al., 2022; Grubišić et al., 2024; Redfield, 1958). Therefore, the cellular ratios of these elements were determined to develop a new medium derived from F/2 for the cultivation of *M. nummuloides*. TN, TP, and Si concentrations in the standard F/2 medium were 12, 1.3, and 14.8 mg/L, resulting in N:P:Si mass ratio of 9:1:11 (molar ratio ≈ 20:1:12) (Fig. 1). In contrast, intracellular quotas of *M. nummuloides* were 42.5 pg N /cell, 7.2 pg P /cell, and 80.7 pg Si /cell, corresponding to an N:P:Si mass ratio of 5.9:1:11.2 and a molar ratio of 13.0:1:12.3, which differ from Redfield ratio (Fig. 1A–1D) (Redfield, 1958).

A previous study found that nitrogen supplementation alone, even without additional phosphorus, significantly promoted phytoplankton growth (Seelen et al., 2025). Accordingly, rather than increasing nitrogen to match the N:P ratio of *M. nummuloides*, phosphorus was fixed at 1.3 mg/L and the nitrogen concentration was adjusted to match the cellular N:P ratio of *M. nummuloides*. In F/2 medium, the N:P ratio is 20—higher than the cellular composition (13)—indicating nitrogen is oversupplied (Fig. 1D). This deviation from the cellular stoichiometry may lead to suboptimal nutrient utilization and increase both production costs and environmental impacts during scale-up (Dortch, 1990; Flynn, 1991). Hence, the development of an optimized medium tailored to the nutrient requirements of *M. nummuloides* was necessary to achieve a balance between product yield, process efficiency, and cost-effectiveness.

### Growth performance of *M. nummuloides* cultivated in modified culture media

**Fig. 2.**
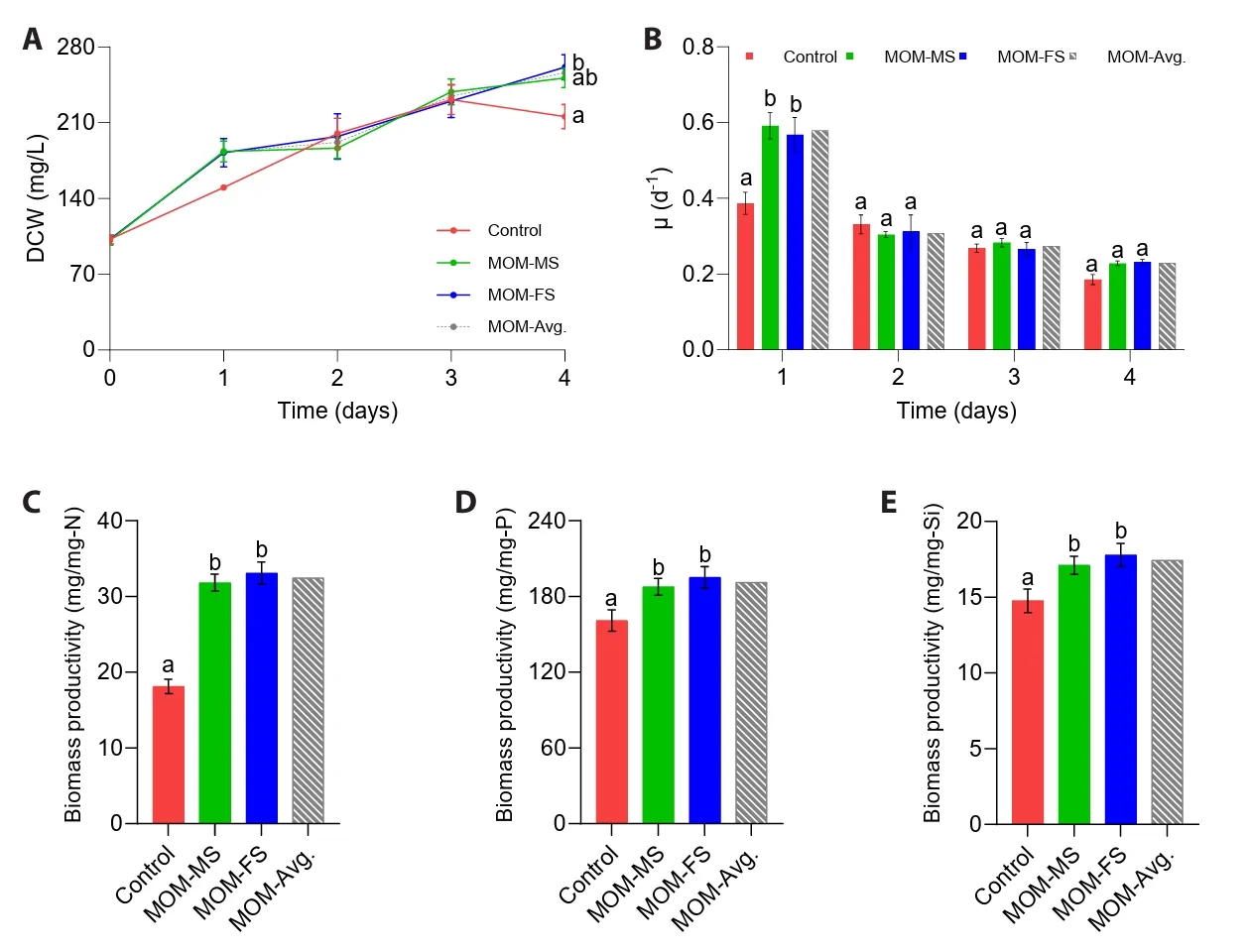
Cultivation performance in F/2 and modified media. (A) Dry cell weight (DCW), (B) Specific growth rate (μ, d^-1^), (C) Biosynthetic biomass yield per unit nitrogen (mg/mg-N), (D) Biosynthetic biomass yield per unit phosphorus (mg/mg-P), and (E) Biosynthetic biomass yield per unit silica (mg/mg-Si). Different letters indicate statistically significant differences (*p* < 0.05). Data represent mean values of three biological replicates, with error bars indicating standard error (SE).

To determine the suitability of the modified media for the cultivation of *M. nummuloides*, growth performance was evaluated based on measurements of DCW, specific growth rate (μ), interval-specific μ, and biomass yield per unit macronutrient (N, P, and Si). The initial DCW of *M. nummuloides* was 99.6 mg/L (Fig. 2A). Despite a 36.13% reduction in nitrogen input relative to F/2, *M. nummuloides* maintained robust growth in MOM-MS and MOM-FS, consistently showing higher DCW than the control (F/2) throughout the cultivation period (Fig. 2A). By day 4, the microalgal biomass of *M. nummuloides* cultivated in MOM-FS medium was 261 mg/L —approximately 1.21-fold higher than F/2 (216 mg/L; *p* < 0.05) (Fig. 2A).

**Fig. 3.**
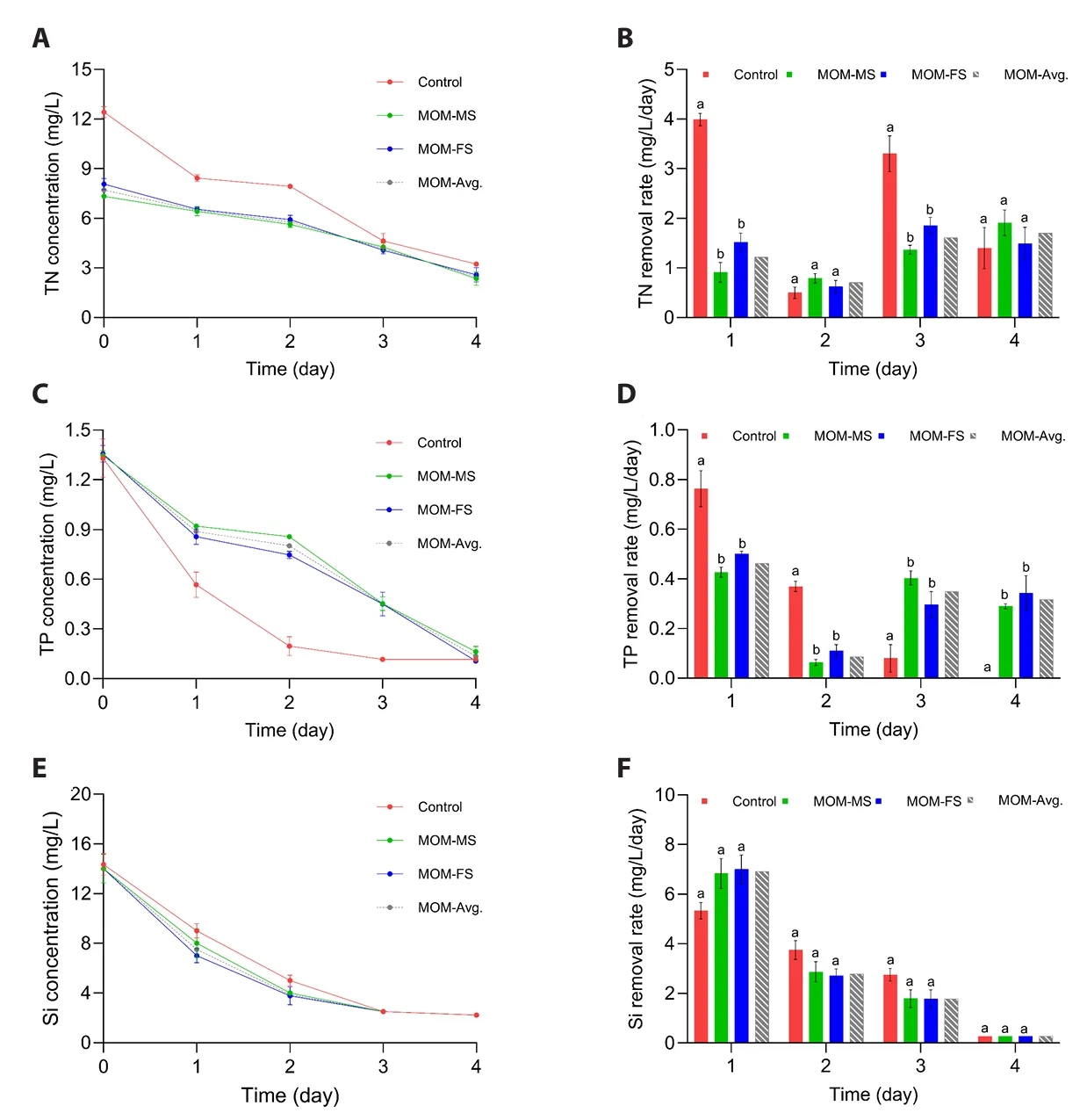
Temporal changes in nutrient concentrations and removal rates over 4 days under different culture media. (A) Total nitrogen concentration, (B) Total nitrogen removal rate, (C) Total phosphorus concentration, (D) Total phosphorus removal rate, (E) Silica concentration, and (F) Silica removal rate. Different letters indicate statistically significant differences (*p* < 0.05). Data represent mean values of three biological replicates, with error bars indicating standard error (SE).

Diatom growth rate has been reported to increase with macronutrient concentration at low levels but decline at higher levels (Grubišić et al., 2024). For example, the growth rate of *Nitzschia* sp. S5 increased as the nitrogen concentration rose from 0 to 3.5 mM, but declined when the concentration further increased to 6–8 mM (Grubišić et al., 2024). In the present study, on day 1, the average specific growth rate (μ) of *M. nummuloides* in MOM-MS and MOM-FS was significantly higher than in the F/2 medium (*p* < 0.05) (Fig. 2B). Consistent with the specific growth pattern, during day 0–1, interval-specific μ of *M. nummuloides* cultured in MOM-MS (0.59 /day) and MOM-FS (0.57 /day) media were higher than in the control (0.39 /day) (Table S1). In the subsequent Day 1–2 interval, the μ of *M. nummuloides* grown in both MOM-MS and MOM-FS media exhibited a marked decline, whereas it decreased more gradually in the control medium. The enhanced growth rate observed in MOM-MS and MOM-FS corresponded to shorter doubling times (τ), decreasing from 3.86 days in the control to 3.06 and 2.98 days, respectively, indicating faster cell proliferation in both modified media. These findings suggest that the relatively high amount of nitrate in F/2 medium may have limited the growth of *M. nummuloides* on days 1 and 2. Considering that at high nitrate levels could be associated with the excessive reactive oxygen species (ROS) generation in algal cells, leading to oxidative stress and impaired cell function (Abassi and Ki, 2022), further investigation is required to validate this hypothesis. By day 4, biomass in the control culture (F/2) decreased markedly, even though nitrogen remained sufficient in the medium, which was likely caused by an imbalance in nutrient stoichiometry. Rapid phosphorus depletion under nitrogen-replete conditions probably triggered P limitation, which in turn led to metabolic inhibition and reduced cell proliferation. In contrast, MOM-MS and MOM-FS maintained balanced nutrient utilization, supporting sustained growth (Fig. 3A–3D).

The biomass yield per unit nitrogen input of *M. nummuloides* cultivated in MOM-MS and MOM-FS media was 31.8 and 33.1 mg/mg-N, respectively, which was higher than F/2 medium (18.1 mg/mg-N) (*p* < 0.05) (Fig. 2C). Similarly, biomass yield per unit phosphorus input increased from 161 mg/mg-P in the control to 188 mg/mg-P in MOM-MS and 195 mg/mg-P in MOM-FS, while silica-use efficiency rose from 14.8 mg/mg-Si in the control to 17.1 mg/mg-Si in MOM-MS and 17.8 mg/mg-Si in MOM-FS (Fig. 2D–2E).

### Nutrient consumption dynamics during cultivation

Phytoplankton generally exhibit increased nutrient uptake rates with increasing nutrient concentrations (Tantanasarit et al., 2013). They can remove nutrients more effectively from the medium when the external supply increases, assimilating them to support binary fission (Tantanasarit et al., 2013). Consistently, TN concentration declined sharply in the control, with a removal rate of 3.99 mg/L/d, but decreased gradually in MOM-MS and MOM-FS media, showing removal rates of 0.91 and 1.52 mg/L/d, respectively, on day 1 (Fig. 3A–3B). After 3 days, no significant differences in TN were detected among the control, MOM-MS, and MOM-FS media. By day 4, TN concentrations in the control, MOM-MS, and MOM-FS were 3.23, 2.36, and 2.58 mg/L, respectively. The R_Xi_ values for N were lower in MOM-MS (4.98 mg-N/g-DCW/d) and MOM-FS (5.28 mg-N/g-DCW/d) compared with the control (10.4 mg-N/g-DCW/d) (Table S3). TP also declined more rapidly in the control than in MOM-MS and MOM-FS on days 1, 2, and 3 (Fig. 3C). The highest R_Xi_ for P was observed in the control (1.38 mg-P/g-DCW/d), while *M. nummuloides* cultured in MOM-MS and MOM-FS removed 1.18 mg-P/g-DCW/d and 1.20 mg-P/g-DCW/d, respectively (Table S3). This phenomenon was associated with a sharp decline in TN. Excessive nitrate concentrations in the medium were likely reduced to intermediate forms, such as NH_4_^+^, NO_2_^-^, and NO within microalgae, leading to overproduction of ROS and cellular stress (Abassi and Ki, 2022; Chen et al., 2009). To scavenge excess ROS, microalgae may activate ATP-dependent pathways, such as autophagy (Laude et al., 2024; Pérez-Martín et al., 2015) and antioxidant enzymatic system (Gauthier et al., 2020). Given that phosphorus is a critical component of ATP synthesis (Su, 2021), an elevated ATP demand consequently intensifies phosphorus requirements, thereby promoting accelerated phosphorus uptake during the early cultivation phase (Fig. 3C). Si consumption patterns were relatively similar across all media, with concentrations declining from 14.1 mg/L at day 0 to 2.22 mg/L by day 4, which remained above the Si-limiting threshold for diatom growth (2 mg/L) (Egge and Aksnes, 1992).

### Fucoxanthin production

**Fig. 4.**
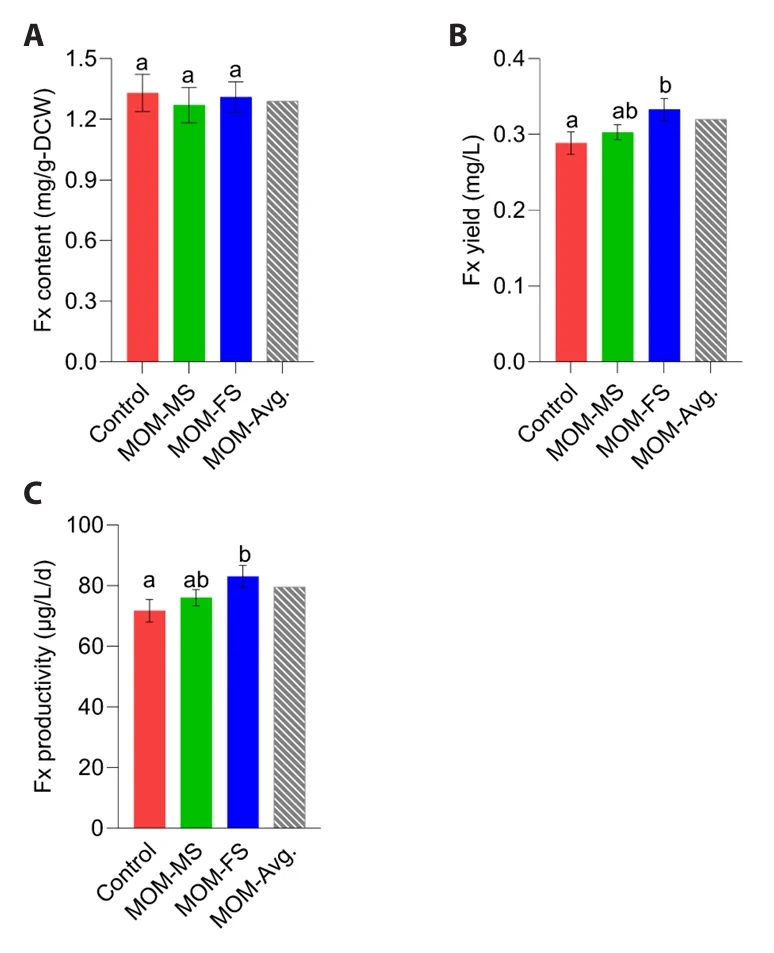
Fucoxanthin production under different culture conditions. (A) Fucoxanthin content (mg/L), (B) Fucoxanthin yield (mg/L), and (C) Fucoxanthin productivity (µg/L/d). Different letters indicate statistically significant differences (*p* < 0.05). Data represent mean values of three biological replicates, with error bars indicating standard error (SE).

Nitrogen concentration is a key determinant of fucoxanthin accumulation (Sun et al., 2019). Nitrate availability may enhance chlorophyll biosynthesis and, in turn, promote fucoxanthin accumulation, as fucoxanthin constitutes a core component of the diatom photosystem (Guo et al., 2016). Therefore, fucoxanthin content can be decreased as the nitrogen source is depleted in the medium (Yoshida et al., 2023). Because TN concentrations were similar among the control, MOM-MS, and MOM-FS on day 4, fucoxanthin content did not differ significantly (Figs. 3A and 4A). After 4 days of cultivation, the highest fucoxanthin yield of *M. nummuloides* was observed in MOM-FS medium with (0.33 mg/L), followed by MOM-MS (0.3 mg/L) and control (0.29 mg/L) (Fig. 4B). Fucoxanthin productivity reached 83 µg/L/d in MOM-FS, representing an ~15.8% improvement over the control (71.7 µg/L/d) (Fig. 4C). Accordingly, an initial TN concentration of 7.9 mg/L in MOM-FS medium appears suitable for supporting *M. nummuloides* growth while maintaining stable fucoxanthin production. Since fucoxanthin content did not differ among media, the higher fucoxanthin yield of *M. nummuloides* in MOM media relative to F/2 is most likely attributable to enhanced biomass productivity (Fig. 4A and 4B).

Fucoxanthin-producing algae are typically classified into two primary groups—macroalgae and microalgae. Compared with macroalgae, microalgae can contain up to 100-fold higher levels of fucoxanthin (Khaw et al., 2022). Owing to their low pigment content and slow growth, macroalgae are generally unsuitable for commercial fucoxanthin production. In this study, the fucoxanthin content of *M. nummuloides* ranged from 1220 to 1330 µg/g-DCW (Fig. 4A), exceeding values reported for macroalgae such as *Himanthalia elongate* (2.8 µg/g) (Lourenço-Lopes et al., 2020), *Laminaria ochroleuca* (4–160 µg/g) (Fernandes et al., 2016; Lourenço-Lopes et al., 2020), and *Saccharina latissimi* (20–120 µg/g) (Fernandes et al., 2016). However, *M. nummuloides* exhibited considerably lower fucoxanthin content and volumetric yield than other microalgae such as *O. aurita*, *P. tricornutum*, and *Nitzschia* (Table S4). For instance, *O. aurita* displayed fucoxanthin contents of 6.39–12.5 mg/g-DCW with volumetric yields of 27.1–79.6 mg/L under nitrogen concentrations of 84–252 mg/L (Xia et al., 2013). *P. tricornutum* reached 12.2 mg/g-DCW and 38.7 mg/L, but required nitrogen inputs as high as 420 mg/L (Yang et al., 2022). *Nitzschia* sp. achieved 18.2 mg/g-DCW and 9.81 mg/L, yet cultivation demanded extremely high nitrogen levels of 3,294 mg/L (Cao et al., 2022). Despite relatively low fucoxanthin yields, *M. nummuloides* biomass holds considerable biotechnological potential owing to its diverse profile of bioactive compounds. For instance, *M. nummuloides* extracts possess strong bioactivities, including antioxidant and anti-inflammatory effects (Cuong et al., 2023), hepatoprotective activity against alcohol-induced liver injury (Kim et al., 2024), and anti-obesity effects in high-fat diet animal models (Yun et al., 2022). In addition to nitrogen concentration, other factors such as the availability of iron and silicate, as well as light intensity and wavelength, can influence fucoxanthin biosynthesis in microalgae (Leong et al., 2022; Palanisamy et al., 2022). Therefore, further studies incorporating biotechnological approaches are needed to elucidate the synergistic effects of these variables and to optimize fucoxanthin production in *M. nummuloides*.

### Bio-silica production

**Fig. 5.**
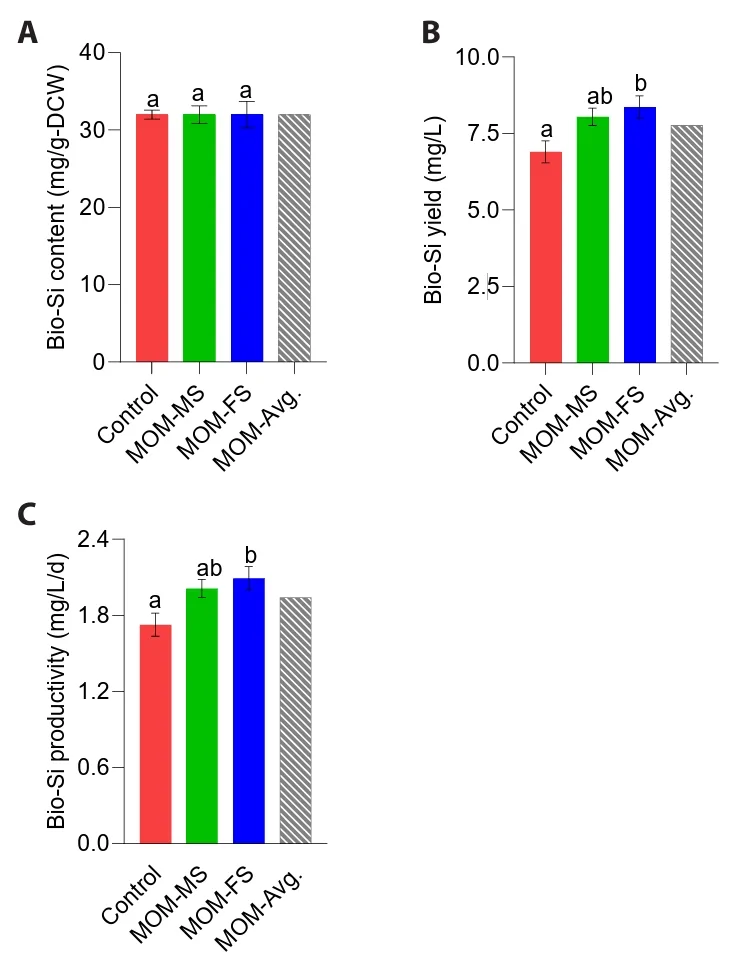
Biosilica production under different culture conditions. (A) Bio-Si content (mg/g-DW), (B) Bio-Si yield (mg/L), and (C) Bio-Si productivity (mg/L/d). Different letters indicate statistically significant differences (*p* < 0.05). Data represent mean values of three biological replicates, with error bars indicating standard error (SE).

Diatom-derived natural biosilica, characterized by its uniform porous nanopattern, hierarchical structure, and abundance of silanol functional groups, has attracted considerable attention for applications in biomedicine, biosensors, gas sensors, cosmetics, and nanomaterials (de Jesus et al., 2025; Kang et al., 2024). The efficient production of bio-silica in diatoms is highly dependent on their growth conditions, such as light intensity, temperature, pH, and nutrient (Panwar and Dutta, 2019). Bio-silica in diatom frustules is synthesized through enzyme-mediated silicification, in which silica polymerases regulate the uptake, transport, and deposition of silicic acid during cell growth (Lim et al., 2023). Thus, diatoms depend on dissolved silicon as an essential substrate for frustule biosynthesis. Given that dissolved Si concentrations did not differ among the control, MOM-MS, and MOM-FS, bio-silica contents of *M. nummuloides* were nearly equivalent (Figs. 4E and 5A). MOM-MS and MOM-FS significantly improved bio-silica productivity compared with the control (*p* < 0.05) (Fig. 5B). Specifically, MOM-FS yielded the highest bio-silica yield and productivity (8.37 mg/L; 2.09 mg/L/d), ~21% greater than the control (6.90 mg/L; 1.73 mg/L/d) (Fig. 5B–5C). Accordingly, the higher volumetric bio-silica yield and productivity observed in MOM-MS and MOM-FS likely resulted from increased biomass accumulation. Silicate, nitrogen, and phosphorus can influence the quality and morphology of bio-silica (Sun et al., 2024). When silicate is insufficient but other nutrients are abundant, diatoms reduce their cell division rate and decrease biosilica mineralization, resulting in thinner frustules. Increasing the phosphate concentration from 10^-6^ M to 100 × 10^-3^ M has been shown to enlarge bio-silica diameters from approximately 50 nm to 600 nm (Sun et al., 2024). Therefore, future research should investigate potential differences in bio-silica morphology between *M. nummuloides* cultured in MOM-FS and those grown in the F/2 medium.

### Cost-effectiveness of MOM-MS and MOM-FS media relative to F/2 medium

Cost-reduction in diatom cultivation has been achieved through partial dilution of the F/2 medium (Kim et al., 2023). The growth rates of *M. nummuloides* were highest in F/2 medium but did not differ significantly from those obtained with F/4 medium, indicating that partial dilution can maintain biomass productivity while reducing nutrient input (Kim et al., 2023). However, such dilution-based strategies primarily aim to preserve growth and generally provide limited improvement in high-value metabolite accumulation. Therefore, rather than simple nutrient dilution, our approach selectively optimizes key F/2 components to sustain biomass production while enhancing biomass-associated fucoxanthin and biosilica accumulation. Nitrogen is a major cost driver in microalgal biomass production, and excessive nitrogen input often leaves residual nitrogen in the medium-raising operational costs; if the spent medium is discharged without a proper nutrient removal process, it can also cause eutrophication in the aqueous environment (An et al., 2024a; Maltsev et al., 2023; Yaakob et al., 2021). The nitrogen source contributes 48.6% of the total cost in F/2 medium, making it the principal target for cost reduction (An et al., 2024b). Moreover, moderate nitrogen limitation is known to stimulate the accumulation of valuable metabolites, including lipids and phosphorus-containing compounds (Xin et al., 2010). Thus, in this study, a cost-effective medium was developed by reducing the nitrogen concentration of the F/2 medium in accordance with the intracellular nutrient stoichiometry of *M. nummuloides*. Unlike other microalgae, diatoms require silicate to facilitate their growth. However, the use of Na_2_SiO_3_·9H_2_O poses a substantial challenge to the economic viability of diatom cultivation at a commercial scale. Na_2_SiO_3_·9H_2_O constitutes 38.7% of the F/2 medium cost (Table 1). Therefore, SiO_2_ was adopted as a more economical silicate source for the cultivation of *M. nummuloides*. The price of composition necessary for the production of F/2 medium was US$30.2 per ton (Table 1). MOM-FS not only enhanced the growth of *M. nummuloides* but also improved cost-effectiveness. By reducing nitrogen concentration and changing silica source, the cost of MOM-FS was reduced by 28.3% relative to the conventional F/2 medium (Table 1). Furthermore, the use of MOM-FS medium can reduce 36.8 and 28.3% fucoxanthin and bio-silica production costs, respectively (Table 2). These results indicate that MOM-FS is a practical, efficient, and economically viable medium for large-scale cultivation of *M. nummuloides* and the co-production of fucoxanthin and bio-silica.

In conclusion, this study demonstrates that species-specific intracellular N:P:Si stoichiometry can guide the development of a cost-effective medium for large-scale diatom cultivation. The MOM-FS medium significantly improved the growth performance of *M. nummuloides* relative to conventional F/2, increasing biomass production by 18.8%, fucoxanthin yield by 10.3%, and bio-silica yield by 20.8%, while reducing medium cost by 28.3%. Collectively, MOM-FS is a practical, efficient, and economically viable medium for large-scale cultivation of *M. nummuloides* and for the co-production of fucoxanthin and bio-silica. Future work should validate MOM-FS across cultivation scales (e.g., outdoor raceways and closed photobioreactors) and include techno-economic analyses to confirm commercial feasibility.

## Figures and Tables

**Table 1.** Nutrient composition of the culture media. The price of each material was established using Sigma-Aldrich's cell culture grade as a basis.

Components	Concentration (mg/L)	Cost (US$/ton)	Concentration (mg/L)	Cost (US$/ton)	Concentration (mg/L)	Cost (US$/ton)
NaNO_3_	75	13	47.9	8.29	47.9	8.29
NaH_2_PO_4_·H_2_O	5	1.22	5	1.2	5	1.22
Na_2_SiO_3_·9H_2_O	30	11.7	30	11.7	-	-
SiO_2_	-	-	-	-	14.8	3.92
NaOH	-	-	-	-	19.7	3.92
Trace metals						
FeCl_3_·6H_2_O	3.15	1.44	3.15	1.44	3.15	1.44
Na_2_EDTA·2H_2_O	4.36	2.29	4.36	2.29	4.36	2.29
CuSO_4_·5H_2_O	0.0098	0.0033	0.0098	0.0033	0.0098	0.0033
Na_2_MoO_4_·4H_2_O	0.0063	0.0014	0.0063	0.0014	0.0063	0.0014
ZnSO_4_·7H_2_O	0.022	0.0069	0.022	0.0069	0.022	0.0069
CoCl_2_·6H_2_O	0.01	0.0064	0.01	0.0064	0.01	0.0064
MnCl_2_·2H_2_O	0.18	0.22	0.18	0.22	0.18	0.22
Vitamins						
Thiamine·HCl	0.1	0.181	0.1	0.181	0.1	0.181
Biotin	0.005	0.107	0.005	0.107	0.005	0.107
Vitamin B_12_	0.005	0.087	0.005	0.087	0.005	0.087
Total cost (US$/ton)	30.2		25.6		21.7	

**Table 2.** Cost-effectiveness of MOM-MS and MOM-FS versus F/2 in fucoxanthin and bio-silica production by *Melosira nummuloides*

Medium	F/2 medium	MOM-MS	MOM-FS
Medium cost (US$/ton)	30.2	25.6	21.7
Max. biomass (mg/L)	215.7	251.4	261.4
US$/kg biomass	140	102	83
Max. fucoxanthin (mg/L)	0.29	0.31	0.33
US$/g fucoxanthin	104	82.4	65.7
Max. bio-silica (mg/L)	6.91	8.33	8.37
US$/g bio-silica	4.37	3.07	2.59

## Supplementary Information

Table S1. Specific growth rates (μ) of *Melosira nummuloides* in different media. Different letters in the same row indicate statistically significant differences (*p* < 0.05).

Table S2. Doubling times (τ) of *Melosira nummuloides* in different media. Different letters in the same row indicate statistically significant differences (*p* < 0.05).

Table S3. Specific rate of substrate removal (R_Xi_) for nitrogen (N), phosphorus (P), and silica (Si) in *Melosira nummuloides* in different media. Different letters in the same column indicate statistically significant differences (*p* < 0.05).

Table S4. Fucoxanthin content (mg/g-DW), yield (mg/L), productivity (mg/L/d), nitrogen concentration (mg/L), and nitrogen-normalized yield (mg-Fx/mg-N) in representative diatom species
