# Supplementary material for: Development of a cost-effective medium for enhanced biomass-associated fucoxanthin and bio-silica yields of diatom (Melosira nummuloides)
Source: J Microbiol. 2026 Feb 28;64(2):e2512005. doi: 10.71150/jm.2512005 (PMC13577039; doi:10.71150/jm.2512005)
Supplement: Supplementary file 1 [file jm-2512005-Supplementary-Table-S1.pdf]

Table S1. Specific growth rates (μ) of *Melosira nummuloides* in different media. Different letters in the same row indicate statistically significant differences ($p < 0.05$).

**Interval (d)**	**Control**	**MOM-MS**	**MOM-FS**
0–1	0.39 ± 0.03^{a}	0.59 ± 0.04^{b}	0.57 ± 0.05^{b}
1–2	0.27 ± 0.06^{a}	0.02 ± 0.03^{b}	0.06 ± 0.06^{b}
2–3	0.15 ± 0.03^{a}	0.25 ± 0.03^{a}	0.17 ± 0.05^{a}
3–4	-0.07 ± 0.04^{a}	0.06 ± 0.02^{b}	0.14 ± 0.03^{b}
0–4	0.19 ± 0.01^{a}	0.23 ± 0.0^{b}	0.23 ± 0.01^{b}
