# Supplementary material for: Development of a cost-effective medium for enhanced biomass-associated fucoxanthin and bio-silica yields of diatom (Melosira nummuloides)
Source: J Microbiol. 2026 Feb 28;64(2):e2512005. doi: 10.71150/jm.2512005 (PMC13577039; doi:10.71150/jm.2512005)
Supplement: Supplementary file 2 [file jm-2512005-Supplementary-Table-S2.pdf]

Table S2. Doubling times (τ) of *Melosira nummuloides* in different media. Different letters in the same row indicate statistically significant differences ($p < 0.05$).

Interval (d)	Control	MOM-MS	MOM-FS
0–1	1.88 ± 0.21^{a}	1.20 ± 0.08^{b}	1.29 ± 0.14^{b}
1–2	3.69 ± 1.14^{a}	16.93 ± 7.99^{a}	5.31 ± 1.40^{a}
2–3	5.24 ± 0.64^{a}	3.11 ± 0.38^{a}	4.85 ± 1.39^{a}
3–4	14.90 ± 0.69^{a}	9.65 ± 2.56^{ab}	6.25 ± 0.97^{b}
0–4	3.86 ± 0.28^{a}	3.06 ± 0.09^{b}	2.98 ± 0.10^{b}
