# Supplementary material for: Development of a cost-effective medium for enhanced biomass-associated fucoxanthin and bio-silica yields of diatom (Melosira nummuloides)
Source: J Microbiol. 2026 Feb 28;64(2):e2512005. doi: 10.71150/jm.2512005 (PMC13577039; doi:10.71150/jm.2512005)
Supplement: Supplementary file 3 [file jm-2512005-Supplementary-Table-S3.pdf]

Table S3. Specific rate of substrate removal (R_{Xi}) for nitrogen (N), phosphorus (P), and silica (Si) in *Melosira nummuloides* in different media. Different letters in the same column indicate statistically significant differences ($p < 0.05$).

Culture medium	Specific rate of substrate removal (R_{Xi})		
	N (mg-N/g-DCW/d)	P (mg-P/g-DCW/d)	Si (mg-Si/g-DCW/d)
Control	10.4 ± 0.34[a]	1.38 ± 0.13[a]	13.8 ± 1.00[a]
MOM-MS	4.98 ± 0.29[b]	1.18 ± 0.02[a]	11.8 ± 1.16[a]
MOM-FS	5.28 ± 0.11[b]	1.20 ± 0.05[a]	11.3 ± 1.11[a]
