# Supplementary material for: Development of a cost-effective medium for enhanced biomass-associated fucoxanthin and bio-silica yields of diatom (Melosira nummuloides)
Source: J Microbiol. 2026 Feb 28;64(2):e2512005. doi: 10.71150/jm.2512005 (PMC13577039; doi:10.71150/jm.2512005)
Supplement: Supplementary file 4 [file jm-2512005-Supplementary-Table-S4.pdf]

Table S4. Fucoxanthin content (mg/g-DW), yield (mg/L), productivity (mg/L/d), nitrogen concentration (mg/L), and nitrogen-normalized yield (mg-Fx/mg-N) in representative diatom species

Species	Medium	Fx yield (mg/L)	Fx productivity (mg/L/d)	TN (mg/L)	Fx per N (mg-Fx/mg-N)	Reference
O. aurita	Modified F/2 medium	79.6	7.96	252	0.316	Xia et al. (2013)
O. aurita	Modified F/2 medium	27.1	2.71	84	0.323	Xia et al. (2013)
P. tricornutum	Modified F/2 medium	32.2	2.68	420	0.077	Yang et al. (2022)
Nitzschia sp.	Zarrouk medium	10.1	1.44	1,648	0.006	Cao et al. (2022)
M. nummuloides	**F/2 medium**	**0.29**	**0.072**	**11.9**	**0.024**	**This study**
M. nummuloides	**MOM-FS**	**0.33**	**0.083**	**7.90**	**0.041**	**This study**
M. nummuloides	**MOM-MS**	**0.30**	**0.076**	**7.90**	**0.038**	**This study**

References

Cao Z, Shen X, Wang X, Zhu B, Pan K, et al. 2022. Effect of nitrogen concentration on the alkalophilic microalga *Nitzschia* sp. NW129-a promising feedstock for the integrated production of lipids and fucoxanthin in biorefinery. *Front Mar Sci.* 8: 830590.

Xia S, Wang K, Wan L, Li A, Hu Q, et al. 2013. Production, characterization, and antioxidant activity of fucoxanthin from the marine diatom *Odontella aurita*. *Mar. Drugs* 11: 2667–2681.

Yang R, Wei D, Pohnert G. 2022. Nitrogen utilization analysis reveals the synergetic effect of arginine and urea in promoting fucoxanthin biosynthesis in the mixotrophic marine diatom *Phaeodactylum tricornutum. Front Mar Sci.* 9: 947726.
